# Lectures on the Exploration and Treatment of Diseases of the Chest

**Published:** 1840-07-04

**Authors:** W. W. Gerhard


MEDICAL EXAMINER.

DEVOTED TO MEDICINE, SURGERY, AND THE COLLATERAL SCIENCES.

No. 27.] PHILADELPHIA, SATURDAY, JULY 4, 1840. [Vol. III.

### LECTURES ON THE EXPLORATION AND TREATMENT OF DISEASES OF THE CHEST.

BY W. W. GERHARD, M. D.

### Lecture VIII.—*Varieties of Pleurisy.*

Besides its simple form, pleurisy presents many varieties which are for the most part connected with various structural alterations of the lungs, or with peculiar symptoms of the disease itself. I have already given a passing notice to several of them; but they require something more, as they constitute the most difficult cases of the disease.

There are other varieties of the disease which differ from the usual form, but at the same time are not connected with an important change in the substance of the lung; that is, latent and chronic pleurisy. These are sometimes closely connected together; thus, latent pleurisy is almost always chronic, but chronic pleurisy is not necessarily latent. I shall first allude to the variety of it which follows a primitive acute pleurisy.

It is difficult in many cases to say what renders an ordinary acute pleurisy chronic; sometimes it is evidently a badly treated case of acute pleurisy, and the inflammation continues, although some of its symptoms may cease. At other times the inflammation has either entirely resisted the ordinary remedies, or it has recurred after having nearly ceased. Both of these varieties present the same symptoms; the physical signs are similar to those of acute pleurisy, but there is evidently an increase in quantity and weight of the effused liquid; hence the prominence of the chest, the displacement of the viscera of the abdomen and thorax, and the flatness of the chest, are all much more decided than in ordinary pleurisy, while the bronchial respiration, as well as the egophony, gradually ceases. I have already stated that the general signs of acute pleurisy, such as the inflammations, fever, and severe pain, may gradually disappear; but the fever is apt to recur, and changes its type, either resembling hectic very closely, or becoming perfectly identical with it. The fever is one of the most troublesome and alarming symptoms of this variety of pleurisy; for in other respects the patient does not suffer in a manner proportioned to the extent or the duration of the effusion. I once saw a patient who had performed the full duties of a sailor, going aloft, &c., with an enormous pleuritic effusion; when he returned from sea, it amounted to two or three gallons. This is an exceptional case; but it is very frequent to find patients who can perform many laborious occupations without much inconvenience: it is generally the case if the dyspnœa be not severe, and you will find that some patients complain of little difficulty of breathing with an extent of pectoral disease which will give rise to great distress in other individuals. The symptoms which so frequently characterize chronic organic diseases, are extremely variable in this variety of pleurisy: these are emaciation, loss of firmness of muscles, harshness and dryness of the skin, and slight œdema of the legs. Sometimes they are nearly as well marked as in tuberculous disease of the lungs,—in other cases they are very slight; hence they constitute a diagnostic sign of the disease, and if you find them well characterized, you will do right to regard the case as one probably complicated with tubercles; if your impression be erroneous, you will soon rectify it, as the symptoms will gradually become more decided in the latter case, and slowly disappear if the pleurisy be followed by recovery.

The diagnosis of chronic pleuritic effusion is often quite impossible without the physical signs, for its symptoms are sometimes nearly similar to those of phthisis. When the physical signs of the disease are present, there is no difficulty in ascertaining it; if it be complicated with tuberculous deposit, the case should be regarded as one of great danger, and your diagnosis is, as we shall afterwards see, much more difficult.

I have already alluded to the prognosis in this variety when speaking of ordinary pleurisy; it is always doubtful, if the effusion be very large, for the liquid then consists nearly of pure pus, and of course the irritation caused by it may be sufficient to produce marasmus, and perhaps deprive the patient of the strength necessary for a cure. The disease may occasionally, though rarely, prove fatal, from the mere obstruction to breathing. The liability of the disease to give rise to secondary tuberculous deposit, after the absorption of the pus, is also to be taken into your account: this forms a variety of the tuberculous pleurisy, in connection with which I shall presently speak of it. There is another way by which chronic pleurisy may terminate fatally,—that is, by producing metastatic abscesses in parenchymatous organs, as the lungs or liver; this result is, however, not common.

The treatment of chronic pleurisy differs so little from that of the acute variety, that I have treated of it at some length in connection with the latter disease. The disease is in both cases essentially the same; but, as it has become chronic, it requires chronic remedies: as a general rule, these should be such as are at the same time antiphlogistic, and favour the absorption of the pus. But in using these remedies, you must not commit a common error, and attempt to force nature through a process which is essentially a slow one; thus, if you subject the patient to what is called a vigorous treatment, you rather impede than favour the cure, and the strength may fail in the attempt. It is on this account that I advise the repeated application of small blisters, warm clothing over a large portion of the body, which is a mild, but powerful means of counter-irritation, and the careful administration of the mercurials and other remedies favouring absorption. Sometimes tonics are necessary in very old pleurisy, as in other diseases in which there is an abundant suppuration; for the strength may fail at the critical point when the largest demands are made upon it. In these cases the essential remedies are the chalybeate preparations, which you may use from time to time, and occasionally either combine or alternate them with the vegetable tonics; but as the influence of the latter is much stronger in restoring the state of the digestive functions than in producing a decided alterative effect upon the general system, they are rather secondary remedies. I have already alluded to the good effects of travelling, and even of a sea-voyage in the treatment of chronic pleurisy. There is another cutaneous tonic and alterative which may be properly combined with them,—that is, stimulating baths, especially the sulphur and salt water baths. These are generally taken at natural sources, by resorting to the sulphur springs or sea-bathing. They are much more powerful, and more safe, taken warm, than cold, especially if you use the artificial baths. But sea-bathing, or bathing in cold sulphur water, is sometimes advisable as a mere tonic, when the patient is simply debilitated, and the inflammation has subsided; they are always remedies which require some caution in their management. In chronic pleurisy it frequently becomes a question whether the operation of paracentesis should be practised. This is, as you well know, one of the most simple operations in surgery, and no one can meet with the least difficulty in performing it,—but, at the same time, it is often very serious in its consequences. There is a rule in surgery which is here strictly applicable; that is, that the exposure of a large suppurating cavity to the air, necessarily excites hectic fever, and sometimes favours the development of secondary abscesses. The chances of recovery are not, therefore, on the whole, increased by the operation,—and it is one which you should not perform, unless it be to relieve excessive dyspnœa, which may in itself be severe enough to threaten life.

### LATENT PLEURISY.

This is another variety of the disease: like all latent inflammations, it is not indicated by the usual functional signs. These are in pleurisy, pain, cough, dyspnœa, and fever, all of which may be either wanting, or so obscure as scarcely to attract notice. When the disease is slight and latent, it passes through its stages without notice, and the patient usually forgets the trifling indisposition under which he may have laboured; it is in this way that adhesions are so frequently formed in the pleuræ of persons who have no recollection of the previous inflammation. When the latent pleurisy is more severe, it gives rise to more decided symptoms; but these are very slow in their progress and formation, and increase very gradually, producing a disturbance of the general system, attended with slow wasting of strength and slight fever, rather than with any symptoms which point decidedly to the local inflammation. A disease which begins in this way is necessarily an obscure one, and may imperceptibly attain a degree of severity which will either render it fatal of itself, or, as is much more frequent, give rise to other disorders, especially of the tuberculous kind. Indeed, many cases of tuberculous pleurisy are in their nature more or less latent; for the peculiarity of latent pleurisy consists merely in the absence of the ordinary local signs; it may or may not be complicated with this morbid deposit.

The diagnosis of latent pleurisy is of course more difficult than that of any other variety of the disease. It depends upon the physical signs of the local mischief, and the evidence of general disorder of the economy. When the disease is attended, as it often is, with considerable effusion, there can be no difficulty in deciding as to its nature, provided all the physical signs can be detected,—that is, the dull or flat percussion, feeble respiration, and egophony: if the friction sound be present, it is of course still more evident. But if the signs be limited to the mere feebleness of respiration and dulness of percussion, you must take care not to confound the disease with an enlargement of the liver, or a chronic consolidation of the lung. As a general rule, however, the physical signs of latent pleurisy are tolerably well marked in all severe cases, when you compare them with those constitutional symptoms which are commonly caused by the disease. These generally pursue the following order:—a patient previously in good or passable health is taken with a slight chill, which is sometimes so short that he is scarcely conscious of its occurrence; this is followed by a slight fever, increasing a little towards the close of the day, but rarely severe enough to destroy the appetite; this is, however, a little diminished, while the thirst and dryness of the skin are rather increased. There is often a slight hacking cough, but the expectoration is altogether or nearly wanting. The strength of the patient is a little enfeebled, but not enough to prevent him from attending to his ordinary business. These symptoms are so slight that most patients are totally unable to localize their disease; this is indeed so difficult that I have known several experienced physicians, who were labouring under this affection, without being able to make a positive diagnosis in their own case.

If you remember, therefore, that latent pleurisy is rarely important, unless it be discoverable by the physical signs,—for in no other case does the effusion take place to any great extent,—you will rarely meet with much difficulty in recognising the disease. The common source of error is in distinguishing between it and pulmonary phthisis, which is sometimes excessively difficult, for the one may often be complicated with the other. This is particularly true of tuberculous pleurisy, in which there is an actual deposit of tubercles, either in the pleuræ or the lungs, and yet the ordinary symptoms of pleurisy are present. I do not wish at present to enter more at length into this matter: it is one of those things which are most difficult to describe; the diagnosis depends upon a number of circumstances, which, in themselves, are unimportant, and acquire value only from their combination.

The prognosis of this form does not differ from that of other varieties of the same disease,—that is, of those which are equally chronic; and except in the cases in which the disease passes into the tuberculous form, it generally terminates favourably: if it be long neglected, however, the disease is sooner or later transformed into pulmonary phthisis.

The treatment of latent pleurisy does not differ in any respect from that of the ordinary chronic forms; and I need not, therefore, repeat what I have already detailed to you.

### SECONDARY AND COMPLICATED PLEURISY.

Pleurisy is secondary to many other diseases: these may be either the affections of the lungs proper, or of the economy in general. When pleurisy occurs during the course of a disease of the lungs, it is most apt to develope itself when the external portions which are nearest to the serous membranes are affected: thus, pneumonia, gangrene, and phthisis, which are the diseases most frequently followed by pleurisy, are often not complicated with it until the disease has advanced from the central parts of the lung, where they generally begin, to the surface; hence the pleuritic stitch or pain may not be felt until a comparatively late period. As a general rule, all affections of the lungs which approach the pleura, will give rise to pleurisy, which is the surest safeguard against perforation of the pleura.

These cases of secondary pleurisy are generally classed with the diseases of the parenchyma with which they are connected; for these are much more important disorders. The treatment is especially directed towards the pleurisy only so far as it is designed to remove pain. In other respects, the same mode of treatment which is proper for the removal of the inflammation of the parenchyma, will usually relieve the disease of the serous membrane. The serous inflammation may occasionally prove more severe than the parenchymatous disorder; thus, there may be but little disease of the substance of the lung, and rather a large effusion into the pleura: this variety is then called pleuro-pneumonia, and it becomes very little else than a pleurisy aggravated by the pulmonary disease.

The tuberculous pleurisy is a disease of some importance: in certain cases it is consecutive to the tuberculous deposit in the parenchyma of the lungs, and is then strictly secondary; in another class of cases the tuberculous deposit takes place in the pleura, and is followed by the inflammation; and in a third the inflammation occurs in an individual who is previously in good health, or at least free from evident tuberculous disease of the lungs, which does not occur until the inflammation has taken place. The first two varieties belong exclusively to the subject of pulmonary phthisis; the latter is rather a cause than a consequence of it,—hence it merits some notice in this place.

The third class, you observe, I divide into two subdivisions,—in the one of them, the tuberculous disease of the lungs occurs after the pleurisy has lasted for some time, or the effusion has perhaps been partially or entirely absorbed. It is difficult to say why a simple pleurisy should be more frequently followed by tubercles than pneumonia, yet such is the fact; or at least there are many cases of pleurisy in which neither attentive observation nor careful reasoning can lead us to suspect the occurrence of tuberculous disease during the active period of the inflammation, although it is developed in its declining stage or at its close. If I were to hazard a theory, I should say that the singular analogy between the irritative fever from pleuritic inflammation with purulent effusion and that resulting from the acute tuberculous disease, shows that there is a singular alliance between the two kinds of morbid action. This explanation, however, even if its correctness were perfectly proved, does not entirely solve the difficulty; but it is certain that the very different ways in which tubercles accompany pleurisy, prove that the mere absorption of pus will not account for it in a large proportion of cases, although the transmission of the purulent fluid through the system must be more or less deleterious, and, like all enfeebling agents, it will break up the constitution, and favour tuberculous diseases.

The second mode in which tubercles seem to arise from pleurisy is probably rather more frequent than that which I have just described; the pleuritic inflammation occurs in healthy individuals, or those who are apparently healthy; and in the serous membrane as well as in the coating of coagulable lymph or false membrane, we find a great number of minute granulations of various size, some barely visible, others of the diameter of half a line or a line, each surrounded by a beautiful net-work of vessels passing to them. The granulations are, as a general rule, most numerous where the vessels are most developed, although this is not invariably the case. In this variety, it would be an abuse of reasoning to conclude that the tuberculous granulations had existed in a latent state, and were followed by the serous inflammation; for they are, for the most part, equal in size, and evidently of extremely recent origin, some of them often appearing in the false membranes, which are necessarily consecutive to the pleurisy. There is, of course, something besides the pleurisy; for all cases of inflammation do not give rise to tubercles, although the exciting causes of the disease, when complicated with them, are the same as of ordinary inflammations. This variety of pleurisy has been little noticed by writers; indeed, you will not, I believe, find that its true value as a cause of tuberculous disease of the parenchyma of the lungs, is any where pointed out.

The varieties of tuberculous pleurisy which are consecutive to pulmonary phthisis, belong more properly to the history of the latter disease than to that of pleurisy proper. The other anomalous products which occasionally take place in the lungs are often complicated with pleurisy; and in a few rare cases you will find that the cancerous or melanotic substance is secreted simultaneously with the serous inflammation: this disease is similar in many respects to tuberculous pleurisy, but possesses little or no practical interest.

